# Comparative analysis of clinical breakpoints, normalized resistance interpretation and epidemiological cut-offs in interpreting antimicrobial resistance of *Escherichia coli* isolates originating from poultry in different farm types in Tanzania

**DOI:** 10.1099/acmi.0.000540.v4

**Published:** 2023-07-14

**Authors:** Ruth Maganga, Emmanuel Sindiyo, Victor Moses Musyoki, Gabriel Shirima, Blandina T. Mmbaga

**Affiliations:** ^1^​ University of Birmingham, Birmingham, B15 2TT, UK; ^2^​ University of Glasgow, Glasgow, G12 8QQ, UK; ^3^​ Kilimanjaro Christian Medical Center/Kilimanjaro Clinical Research Institute, PO Box 2236, Moshi, Tanzania; ^4^​ The Nelson Mandela African Institution of Science and Technology, PO Box 447, Arusha, Tanzania; ^5^​ Department of Medical Microbiology, University of Nairobi, PO Box 19676-00202, Nairobi, Kenya

**Keywords:** breakpoints, antimicrobial resistance, susceptibility, resistance interpretation, *Escherichia coli*, inhibition zone diameter, normalized resistance interpretation, epidemiological cut-offs

## Abstract

**Introduction.:**

Existing breakpoint guidelines are not optimal for interpreting antimicrobial resistance (AMR) data from animal studies and low-income countries, and therefore their utility for analysing such data is limited. There is a need to integrate diverse data sets, such as those from low-income populations and animals, to improve data interpretation.

**Gap statement.:**

There is very limited research on the relative merits of clinical breakpoints, epidemiological cut-offs (ECOFFs) and normalized resistance interpretation (NRI) breakpoints in interpreting microbiological data, particularly in animal studies and studies from low-income countries.

**Aim.:**

The aim of this study was to compare antimicrobial resistance in *Escherichia coli* isolates using ECOFFs, CLSI and NRI breakpoints.

**Methodology.:**

A total of 59 non-repetitive poultry isolates were selected for investigation based on lactose fermentation on MacConkey agar and subsequent identification and confirmation as *E. coli* using chromogenic agar and *uidA* PCR. Kirby Bauer disc diffusion was used for susceptibility testing. For each antimicrobial agent, inhibition zone diameters were measured, and ECOFFs, CLSI and NRI bespoke breakpoints were used for resistance interpretation.

**Results.:**

According to the interpretation of all breakpoints except ECOFFs, tetracycline resistance was significantly higher (TET) (67.8 –69.5 %), than those for ciprofloxacin (CIPRO) (18.6 –32.2 %), imipenem (IMI) (3.4 –35 %) and ceftazidime (CEF) (1.7 –45.8 %). Prevalence estimates of AMR using CLSI and NRI bespoke breakpoints did not differ for CEF (1.7 % CB and 1.7 % CO_WT_), IMI (3.4 % CB and 4.0 % CO_WT_) and TET (67.8 % CB and 69.5 % CO_WT_). However, with ECOFFs, AMR estimates for CEF, IMI and CIP were significantly higher (45.8, 35.6 and 64.4 %, respectively; *P*<0.05). Across all the three breakpoints, resistance to ciprofloxacin varied significantly (32.2 % CB, 64.4 % ECOFFs and 18.6 % CO_WT_, *P*<0.05).

**Conclusion.:**

AMR interpretation is influenced by the breakpoint used, necessitating further standardization, especially for microbiological breakpoints, in order to harmonize outputs. The AMR ECOFF estimates in the present study were significantly higher compared to CLSI and NRI.

## Data Summary

Data used in the current study are provided in the supplementary material. Sheet 1 (EUCAST and MYDATA) presents a breakdown (in proportion) of the isolates per zone diameter. Additionally, it provides data on the proportion of EUCAST reference isolates per zone diameter. Distribution curves were generated from both sets of data in sheet 1 using EUCAST distribution curves as a reference. Sheet 2 (Molecular and Phenotypic results) shows 74 isolates that were shipped to Glasgow for further analysis including 59 isolates that were positive for *Escherichia coli* following *uidA* PCR, which were subsequently used for downstream analysis to produce distribution curves that were then compared to reference EUCAST distribution.

## Introduction

Antimicrobial resistance (AMR) has emerged as a major public health concern due to the rapidly diminishing efficacy of antimicrobial therapy [1]. Phenotypic approaches have long been acknowledged as the gold standard for detecting resistance both in clinical and in microbiological contexts [2]. Despite the prominence of molecular approaches, phenotypic techniques remain crucial for quantifying resistance and sensitivity, whereas genotypic techniques are useful for predicting resistance and determining resistance mechanisms [3]. Susceptibility testing is commonly used in clinical settings to determine how bacteria respond to empirical therapy, and one of the most popular criteria for determining if an antimicrobial is effective is estimating the lowest dosage at which microbial growth may be suppressed [3–5]. Well-known susceptibility metrics include inhibition zone diameter (IZD) and minimum inhibitory concentration (MIC) [5]. In the MIC assessment, micro-organisms are cultured in liquid media in the case of broth microdilution in slots with varying concentrations of the antimicrobial agent under study, ranging from high to low, and the lowest MIC score inhibiting growth of microbes is then determined [5]. IZDs are usually measured on solid media, in which antimicrobial discs of known concentrations are placed on plates streaked with bacteria culture, the antimicrobials diffuse away from the disc, generating a concentration gradient that inhibits bacterial growth at a measurable radius from the disc, resulting in a zone of inhibition [5, 6]. The wider the inhibition zone, the easier it is to treat the population of micro-organisms being examined.

Selecting a breakpoint guideline to adopt is largely driven by the objective of the analysis [7, 8]. AMR in clinical and microbiological contexts differs greatly, as do the functions of breakpoints [8]. In clinical settings, the term ‘resistance’ refers to a condition in which a patient’s clinical recovery requirements are not met while receiving the correct antimicrobial dosage [8–10]. By contrast, in a microbiological context, resistance pertains to the mechanisms that make an isolate less susceptible to an antimicrobial agent compared to other isolates of the same species [2, 10]. Therefore, microbiological breakpoints distinguish isolates that have evolved resistance through mutations or horizontal gene transfer from wild-type isolates, independent of whether the degree of resistance is clinically significant [7]. The current investigation considers wild-type organisms as those with ‘typical’ susceptibility patterns to antibiotics [11]. These would be regarded as those not having acquired resistance genes or genetic changes, making them sensitive to antimicrobial agents [11]. There is a significant knowledge gap and a lack of understanding of the application of the different breakpoints in the literature. In recent literature, clinical and microbiological breakpoints are often used interchangeably [7, 8], leading to confusion and a reduction in the relevance of the research involved. Even though EUCAST (European Committee on Antimicrobial Susceptibility Testing) epidemiological cut-offs (ECOFFs) and CLSI (Clinical & Laboratory Standards Institute) breakpoints strive to strike a balance between clinical relevance (e.g. application of pharmacokinetic/pharmacodynamic principles in establishing the breakpoints) and the need to identify emerging resistance, ECOFFs generally maintain that organisms found in the wild-type distribution (susceptible population) have a low likelihood of clinical treatment failure [12]. This may translate into lower breakpoints for EUCAST ECOFFs compared to CLSI, leading to a broader categorization of isolates as susceptible. Erroneous classification of certain isolates as susceptible based on EUCAST breakpoints could theoretically increase treatment failure rates [13]. Despite lack of consensus in the use of breakpoints in the literature, ECOFFs remain the most widely used breakpoints in microbiological research, while CLSI breakpoints are the standard in clinical settings [7–10]. EUCAST ECOFFs were developed in Europe [7], while CLSI breakpoints were developed in the USA [7, 11], although both breakpoints are now universally acknowledged. Recent research has offered a novel approach for establishing breakpoints that employs normalized resistance interpretations (NRIs) to address the EUCAST ECOFF constraints [12–14]. The method involves entering MIC zone sizes of a set of isolates into a spreedsheet and calculating the distribution, smoothing the distribution using rolling means, identifying the peak of the smoothed distribution and calculating the estimated total number of wild-type (WT) observations [12]. The distribution of percentage, cumulative percentage and probit values of the WT observatons are then calculated, and the slope and the intercept of the best-fit line of the probit values versus zone size are determined using a least squares method [12]. The mean and standard deviaton of the normalized WT distribution are then calculated, and the epidemiological cut-off values are set at the mean minus 2.5 times the standard deviation [12]. The functional peak serves as a reference point for determining the portion of the distribution that corresponds to WT isolates and contributes to setting cut-off values/breakpoints for resistance interpretation [12]. A functional peak is generally established after a putative peak has been identified and modulated according to protocol conditions stipulated by Kronvall and Smith [12] using the NRI method. However, one major flaw of this method is that it assumes that the WT observations are symmetrically distributed around the peak which may not hold true in all cases [14–16]. Moreover, the accuracy of the NRI method can be influenced by the size of the dataset used for analysis [12, 14, 16]. Small sample sizes may lead to less precise estimates of the parameters such as the functional peak and standard deviation, potentially affecting the reliability of the interpretation [12, 14, 16]. However, the generated bespoke breakpoints allow for laboratory-specific strain classifications of WT and non-wild-type (NWT) strains [12–14]. The method allows for reconstruction of the normalized peak for MIC or IZD distributions as long as resistance is not developed in the WT population [12, 16]. Tiny variations in zone diameters including low-level resistance in the WT populations can, therefore, be identified even within populations considered primarily susceptible by traditional interpretations (EUCAST and CLSI) [12, 16]. Furthermore, novel forms of resistance can also be detected, increasing antibiotic susceptibility testing sensitivity and precision [13]. By normalizing the data, the technique improves comparability between laboratories and minimizes the effect of variability on resistance interpretation [13]. Consequently, standardization of breakpoints is possible in certain datasets, provided reproducibility and precision are demonstrated [13, 14].

Despite developments in microbiological breakpoints, research into how these breakpoints perform when applied to data from low-income countries is sparse. There is still a dearth of knowledge concerning the validity of prevalence estimates of data from low-income countries employing these breakpoints. EUCAST ECOFFs and CLSI breakpoints are primarily generated with data from developed countries [9]. Moreover, since most of the data used to generate the ECOFF reference distribution are of human origin [9], the underlying breakpoints are unlikely to provide an accurate framework for evaluating data from animals or the environment.

The present study evaluated the prevalence of AMR in four types of poultry farms in Moshi and Arusha, Tanzania, using CLSI breakpoints, ECOFFs, and bespoke thresholds generated from NRIs. The study also aimed to ascertain whether the selected breakpoint has any bearing on resistance prevalence predictions.

## Methods

### Study design and location

This study was part of a broader prospective cross-sectional study conducted in Arusha and Moshi districts in Tanzania whose aim was to determine whether different poultry husbandry systems were associated with varying degrees of AMR among poultry populations. The initial study collected 746 samples out of a target of 800 from four different farm types, with ten cloacal swabs collected per farm type. These data were collected from selected wards, with ten selected from each district, Arusha and Moshi. In the context of this study, wards refer to administrative subdivisions or smaller geographical areas within the districts of Arusha and Moshi, which are used for local governance and representation. Taking budgetary considerations into account, 74 plate sweeps were shipped to the One Health Research in Bacterial Infectious Diseases (ORHBID) laboratory, located at the Institute of Biodiversity, Animal Health and Comparative Medicine, University of Glasgow, for analysis. Of the 74 plate sweeps, 74 isolates which exhibited successful growth following subculture on MacConkey agar were selected for the present study, with each isolate representing each plate.

### Identification of antimicrobial-resistant lactose-fermenting coliforms on MacConkey agar

Using a modified breakpoint plate method described by Caudell [17], we assessed the total population of coliforms and resistant coliforms present on MacConkey agar with and without antimicrobials [15]. Interpretation of resistant coliforms to the selected antimicrobial drugs at defined concentrations was conducted according to CLSI 2016 guidelines. Coliforms formed pink to red colonies, while other lactose-intolerant Gram-negative bacteria formed pale white colonies. Frozen (−80 °C) cloacal swab samples were thawed overnight at 2 °C for isolation. Following homogenization, 50 µl of each sample was added and vortexed with 450 µl of maximum recovery diluent (MRD; Oxoid Thermofisher). Plating was performed on plain MacConkey plates withiout antibiotics and MacConkey plates supplemented with antimicrobial agents using a spiral plater (Spiral System) programmed to dispense 50 µl of the mixture in a logarithmic dilution. Coliforms were enumerated using the spiral plater grid technique at Kilimanjaro Clinical Research Institute (KCRI) after incubation on plain MacConkey agar and MacConkey agar with antimicrobials. Each plate was mapped with a grid, placed on a level surface and adjusted so that the grid’s centre corresponded to the plate’s centre on the viewer. Colonies were counted from the outer border of each section into the centre, allowing the bacterial concentration to be estimated.

### Collection and storage of plate sweeps

Coliform bacteria plate sweeps were obtained from plain MacConkey agar plates and subsequently preserved at −80 °C. Two vials of plate sweeps were collected from each plate, with both vials subjected to storage in a preservation medium consisting of MRD media and 15 % glycerol. One vial was stored at −80 °C and retained in Tanzania for future reference (archived), while the second vial was temporarily stored at −80 °C, awaiting shipment to the OHRBID laboratory at Glasgow University (aliquot used in the present study). To ensure preservation during transportation, the frozen plate sweeps were shipped using dry ice. The primary objective of this shipment was to facilitate further analysis and investigation at the aforementioned laboratory.

### Phenotypic identification of *Escherichia coli* using chromogenic agar

At the OHRBID laboratory, cloacal swabs were thawed overnight at 2 °C and 50 µl of the sample was homogenized with 450 µl of MRD. The mixture was vortexed, and 50 µl was plated on MacConkey agar with a spiral plater (Spiral System) and incubated at 37 °C. Pink lactose-fermenting colonies were inoculated on Luria-Bertani broth (Oxoid) and incubated at 37 °C for 24 h. Pure culture (50 µl) was inoculated on chromogenic agar (CHROMagar ECC; Sigma Aldrich), spread evenly using a sterile L-shaped spreader (VWR; catalogue number 6121560P) and incubated for 24 h at 37 °C. Phenotypic blue colonies indicated the presence of *E. coli* isolates, and selected isolates were confirmed via quantitative *uidA* PCR as described in the section below on comfirmation of *E. coli* species

### Reference strains

As part of this study, reference strains originating from dogs were obtained from the University of Glasgow’s Veterinary Diagnostic Services laboratory for subsequent analysis. Identities of the strains were confirmed using API 20E strips (API system by bioMérieux, available at https://www.biomerieux.co.uk/product/apir-id-strip-range). A positive *E. coli* control and a negative *Klebsiella* species control were used for both genotypic and phenotypic confirmation of *E. coli* isolates. These two reference isolates were resistant to all antimicrobial agents used in this study.

### Molecular detection of *E. coli* using quantitative *uidA* PCR

#### DNA extraction

DNA extraction was conducted using a QIAamp DNA mini-Kit (Qiagen). Isolates from CHROMagar were resuspended in 1 ml Luria-Bertani media (VWR) and 50 µl was processed according to the manufacturer’s instructions provided with the QIAamp DNA mini-Kit. DNA concentrations were determined using the NanoDrop (NanoDrop-2000 Spectrophotometer; NanoDrop Technologies).

#### Confirmation of *E. coli* species using *uidA* PCR

Real-time quantitative PCR (RTqPCR) was performed using the Rotor gene system (Applied Biosystems) to identify the *uid*A gene, an 1809 bp gene expressed by all *E. coli* bacteria. The *uid*A RTqPCR primers and probe used for detection were as described by Frahm and Obst [16]. The probe was labelled with 56-FAM as a reporter fluorescent dye at the 5′ end and the 3′ end with BHQ_1 as the quencher dye. Reactions for *uid*A RTqPCR were performed as described by Frahm and Obst [16]. The RTqPCRs were performed in a 15 µl reaction volume using 2× Quantitect Probe PCR master mix (Qiagen), 0.4 µM of each primer, 0.2 µM of probe (Integrated DNA Technology) and 5 µl of template DNA from presumptive *E. coli* isolates. PCR cycling conditions consisted of an initial denaturation step at 95 °C for 2 min, followed by 45 cycles of denaturation at 95 °C for 5 s and annealing/extension at 60 °C for 5 s.

#### Culture and susceptibility testing using disc diffusion test

Antimicrobial susceptibility testing (AST) was conducted using a standardized disc diffusion technique [17]. *E. coli* was tested against four [4] antimicrobial agents at standard disc quantity according to EUCAST recommendations, i.e. ceftazidime (30 µg), ciprofloxacin (5 µl), imipenem (10 µg) and tetracycline (30 µg). The procedure involved diluting the culture suspension with distilled water to a density of 0.5 MacFarland. Mueller Hinton agar was poured in plates (90 mm in diameter, 4–6 mm in depth). Prior to inoculation, the plates were air-dried for about 30min. Bacterial suspensions at 0.5 MacFarland were streaked evenly across the surface of the medium with a plate spreader (VWR; catalogue number 6121560P). After drying for 3–5 min, the four antimicrobial discs were placed on the agar surface using a sterile forceps and gently pressed down to ensure contact. The plates were incubated at 37 °C under aerobic conditions. After overnight incubation, the zone diameters were measured on the reverse side of the culture plate using a vernier calliper.

#### Data analysis

The IZD for each antimicrobial agent tested was assessed using breakpoints, CB, ECOFFs or wild-type bespoke cutoff (CO_WT_) values based on NRI to identify the prevalence of susceptible and resistant isolates. The clinical breakpoints (CBs) were determined using the 2016 CLSI guideline, ECOFFs were generated using EUCAST guidelines [11]. Since ECOFFs for 30 g tetracycline were unavailable on the EUCAST website, the distribution of tigecycline, a member of the same antimicrobial class as tetracycline with the requisite concentration, was substituted for visual comparison purposes. The focus was on comparing the distribution patterns between the current study and the mentioned antimicrobial class reference distributions, in order to assess the similarity or dissimilarity with our data. However, it is important to note that the breakpoint for tigecycline was not used in this analysis to ensure accuracy and avoid potential misinterpretations. All calculations of the CO_WT_ were conducted according to specifications in a published protocol by Kronvall and Smith [12] using a spreadsheet provided by the authors (European patent No. 1 383 913, US Patent No. 7,465,559; https://doi.org/10.1111/apm.12624). The IZD histograms and CO_WT_ values for each compound were computed using the provided spreadsheet. Additionally, the construction of the functional peak was generated following the steps outlined by Kronvall and Smith [12], utilizing an automated sheet available online at https://doi.org/10.1111/apm.12624. The prevalence of resistance among poultry *E. coli* isolates from Tanzania was calculated using three distinct thresholds: CB, ECOFFs and CO_WT_.

## Results

### Phenotypic and molecular detection of *E. coli*

Out of 74 plate sweeps that were shipped to the University of Glasgow for further analysis and subsequently cultured on MacConkey agar, a total of 74 isolates (one isolate per plate sweep) were successfully cultivated. Following subculture on CHROMagar from MacConkey agar, 72 isolates displayed blue colonies, indicating a presumptive identification as *E. coli.* However, subsequent confirmation using *uidA* PCR revealed that out of these 72 isolates, 59 were confirmed to be *E. coli* (data available in Table S1: Molecular_Phenotypic_results, available in the online version of this article).

### Susceptibility testing

**Fig. 1.**
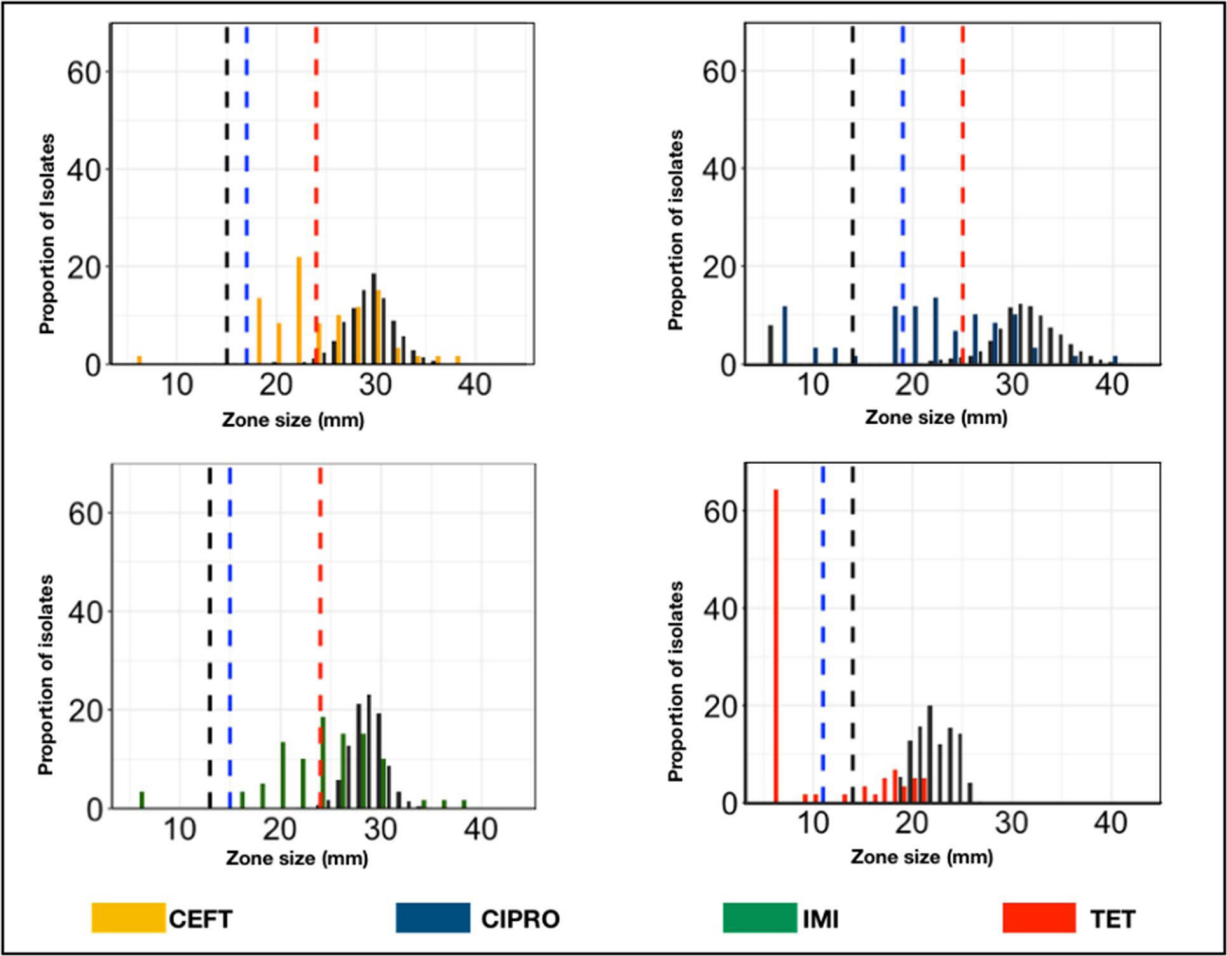
Distribution of inhibition zone diameters (IZDs) produced by 30 µg ceftazidime, 5 µg ciprofloxacin, 10 µg imipenem or 30 µg tetracycline discs against *Escherichia coli *. The coloured bars indicate results for *E. coli* from poultry cloacal swabs from Tanzania (*n*=59). The distribution of IZDs from *E. coli* isolates from EUCAST data is shown in black bars [*n*=11 875, 36 774, 4600 and 326 for ceftazidime (CEF), ciprofloxacin (CIPRO), imipenem (IMI) and tetracycline (TET), respectively]. Dashed lines represent CB (blue), ECOFFs (red; not available for TET) and CO_WT_ (black) based on normalized resistance interpretation of the data from Tanzanian poultry. Data used in plotting the distribution graphs can be found in Table S1: Distributions.

**Fig. 2.**
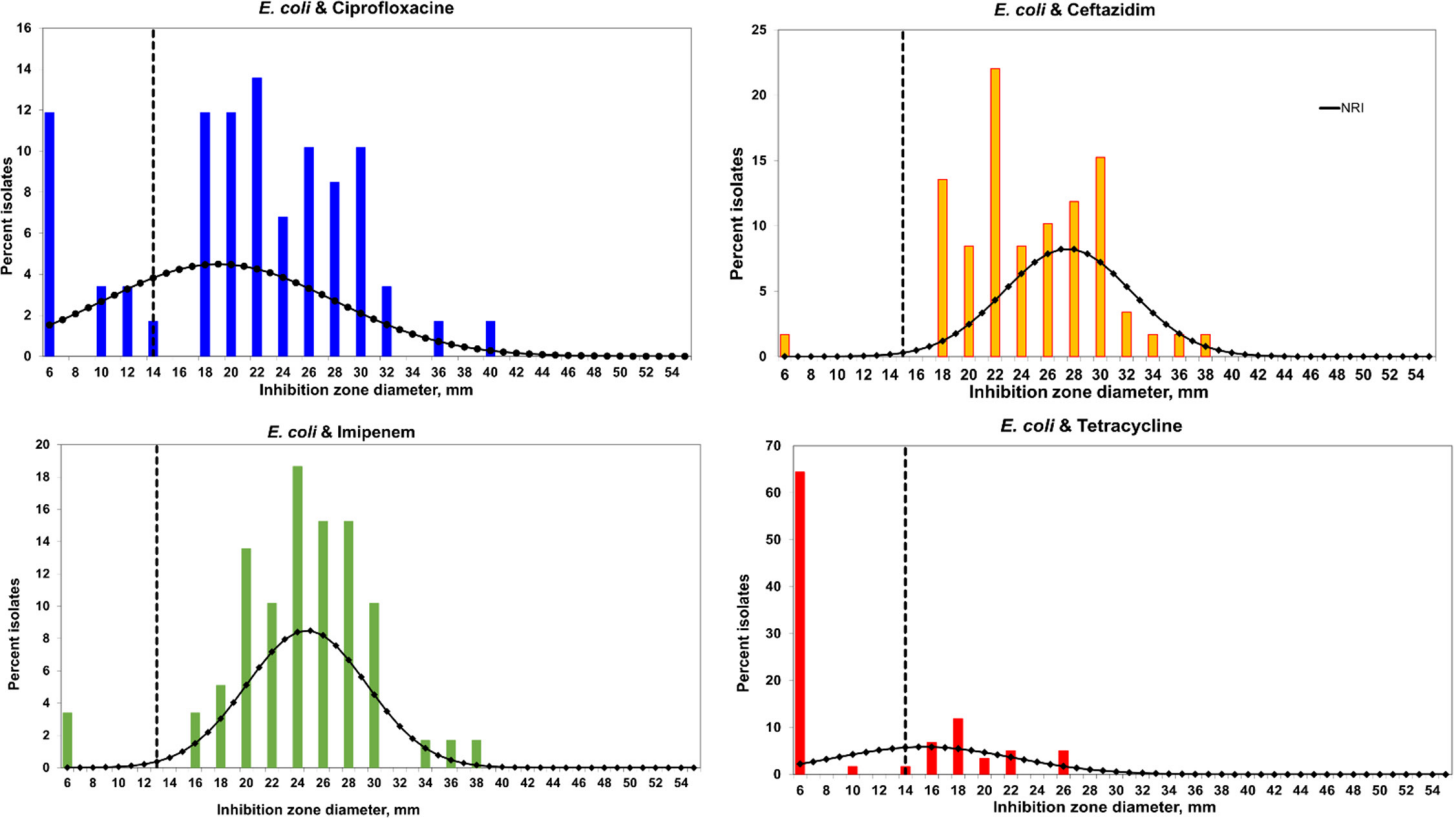
Bar graphs illustrating the inhibitory zones formed by discs containing 30 g ceftazidime, 5 g ciprofloxacin, 10 g imipenem or 30 g tetracycline. The four-point rolling mean is represented by the continuous black curve, while the wild-type cutoff (CO_WT_) is generated from the data using the normalized resistance interpretation method. Graphs were prepared in Microsoft Excel, using the spreadsheet provided by P. Smith, W. Finnegan, and G. Kronvall (European patent No. 1383913, US Patent No. 7,465,559).

In line with the objective of the current study, which seeks to compare resistance interpretation based on three breakpoints, we began by visualizing the range of IZD values collected per antimicrobial as seen in Tables 1 and S1: Distributions. For each antimicrobial class, there was at least one isolate that exhibited a zone diameter of 6 mm. Following that, the distribution of IZD values was examined using the NRI approach and visualized across all antimicrobial classes, as illustrated in Fig. 1. The approach allowed the determination of the mean zone size and standard deviation (sd) for WT isolates, as well as CO_WT_ for each compound, as depicted in Table 1 and normalized histograms in Fig. 2. The estimated sd values for ciprofloxacin, imipenem and ceftazidime IZDs exceeded the recommended limit of 4 mm [18], except for tetracycline (Table 1).

**Table 1.** The functional peak, standard deviation of the functional peak and cut-off values for WT were observed in a range of inhibition zone diameters and output by normalized resistance interpretation (CO_WT_)

Antimicrobial	Range (mm)	Functional peak (mm)	sd (mm)	CO_WT_ (mm)
Ceftazidime	6–38	29.0	4.8	15
Ciprofloxacin	6–40	26.5	5.1	14
Imipenem	6–38	24.5	4.7	13
Tetracycline	6–21	18.0	2.1	14

### Comparison of EUCAST reference data and Tanzanian poultry data

The distribution of IZD values for *E. coli* isolates (Table S1: Distributions) obtained from poultry in Tanzania showed a noticeable shift towards lower IZD values across all antimicrobial agents tested. This shift in values, as illustrated in Fig. 1, indicates reduced susceptibility levels compared to the reference data provided by EUCAST. Specifically, the CB for tetracycline was lower than the CO_WT_. While the clinical breakpoints deviated from the CO_WT_ by 5 mm or less for all compounds, the ECOFFs were significantly higher than the corresponding CO_WT_ values. The wild type cut-offs (CO_WT_) were lower than CB and ECOFFs for all antimicrobials except tetracycline (Fig. 1).

### Estimation of the prevalence of AMR in *E. coli* from Tanzanian poultry based on ECOFFs, CB and CO_WT_

There was no statistically significant difference in the prevalence estimates of ceftazidime, imipenem, ciprofloxacin and tetracycline resistance when interpretation was conducted using the CO_WT_ cut-off and clinical breakpoints (CB) (χ^2^=1.29, d.f.=3, *P*>0.05). However, when comparison of the interpretations of the ECOFFs with the other two breakpoints was conducted, a significantly higher prevalence of resistance was observed for ECOFF values, except for tetracycline where breakpoint values were unavailable for the desired concentration. This difference was statistically significant, as presented in Table 2 (χ^2^=23.91, d.f.=4, *P*<0.05). The proportions of susceptible isolates determined by CB, CO_WT_ and ECOFF values were not significantly different (χ^2^=2.830, d.f.=4, *P*>0.05) for CEF, CIPRO and IMI, as presented in Table 2.

**Table 2.** The proportion of resistant (R), non-wild-type (NWT), susceptible (S) or wild-type (WT) *Escherichia coli* isolates from poultry cloacal samples from Tanzania determined by CB, ECOFFs and the CO_WT_ according to normalized resistance interpretation

Antimicrobial	CB	S (%)	R (%)	ECOFF (mm)	WT (%)	R (%)	CO_WT_ (mm)	WT (%)	NWT (%)
Ceftazidime	17	98.3	1.7	24	54.2	45.8	15	98.3	1.7
Ciprofloxacin	19	67.8	32.2	25	35.6	64.4	14	81.4	18.6
Imipenem	15	96.6	3.4	24	64.4	35.6	13	96.0	4.0
Tetracycline	11	32.2	67.8	–	–	–	14	30.5	69.5

## Discussion

The aim of this study was to examine whether AMR estimates varied depending on the AMR breakpoint used. In contrast to clinical breakpoints, which define resistance as the likelihood of treatment failure, epidemiological cut-offs and bespoke normalized resistance interpretive breakpoints use microbiological criteria to define resistance [2, 8, 10]. Previous studies have used these breakpoints to interpret resistance [6–20]. The current study compared prevalence estimates of resistant *E. coli* isolates based on the epidemiological cut-offs (ECOFFs), CLSI break-points (CBs) and NRI bespoke breakpoints (CO_WT_). Prevalence estimates of ceftazidime, imipenem and tetracycline resistance based on CO_WT_ and CB did not differ significantly; however, ECOFF values for ceftazidime and imipenem resistance were significantly higher. Ciprofloxacin resistance varied significantly across all three breakpoints. However, ECOFFs generated the highest prevalence estimates of ciprofloxacin resistance, followed by CB, and the lowest estimates were generated by CO_WT_. Finding resistance to carbapenem (imipenem), third-generation cephalosporin (ceftazidime) and fluoroquinolone (ciprofloxacin) in poultry is alarming as these antimicrobials are listed as World Health Organisation (WHO) Critically Important Antimicrobials. Third - generation cephalosporins are designated as Highest Priority Critically Important Antimicrobials (HP CIAs) [20]. Additionally, carbapenems and third-generation cephalosporins are rarely used in livestock production in Tanzania [21, 22], making their resistance presence a cause for concern.

Clinical breakpoints (CB) and CO_WT_ breakpoints did not differ significantly in their interpretations of resistance to antimicrobial agents, especially for ceftazidime, imipenem and tetracylines, an observation that differs from the observations of Dias *et al*. [18, 23], where AMR prevalence estimates varied according to the thresholds used [18]. NRI may, however, interpret values as susceptible if (low-level) resistance is prevalent in a dataset [13]. Furthermore, the NRI method has a fundamental flaw in that cutoffs generated from small datasets may not be accurate or representative of a larger population [18]. For instance, three antimicrobials in our data exceeded the allowable standard deviation [18], which necessitates caution when interpreting the results of our study. Small datasets are more susceptible to outliers and random variation, which can lead to high sd values and high variability in NRI results [18]. The present study used a small dataset. As a result, meaningful trends and accurate interpretations are constrained. Additonally, there were several distributions in our study that were bimodal rather than unimodal. Since the NRI technique estimates CO_WT_ values based on the distribution’s highest peak assuming a normal distribution, the significance of the second peak on lower IZD scores is likely to be overlooked, despite it indicating the presence of an intermediate population. To fully understand this phenomenon, larger datasets are needed [11, 24].

The prevalence of tetracycline resistance based on the current dataset was higher than that of other antimicrobials, while ceftazidime and imipenem resistance were low, and ciprofloxacin resistance was moderate. The results were consistent with previous research conducted in the northern part of Tanzania where similar occurrences of tetracycline resistance were found [21, 22, 25]. One of the underlying driving factors is the widespread use of tetracycline in poultry production in northern Tanzania [26, 27]. On the other hand, ECOFF and CB predicted higher estimates of AMR compared to CO_WT_. However, our findings do not align with previous studies conducted in the same districts. Discrepancies may have arisen due to different methodologies used. Contrary to our finding, Hamis *et al*. [22, 28] used both the Kirby–Bauer method and clinical breakpoints and found a significantly higher prevalence of ciprofloxacin resistance. Rugumisa *et al*. [25, 29] used the breakpoint plate method and clinical breakpoints and found lower rates of ciprofloxacin resistance. As our study and the referred studies were conducted on different poultry populations, the results may also indicate that ciprofloxacin resistance varies by population.

Resistance to imipenem and ceftazidime were observed in poultry *E. coli* isolates. However, these agents are rarely used in poultry production in Arusha, according to a qualitative survey on antimicrobial use published by Sindiyo *et al*. in 2018 [26]. Consequently, it was not anticipated that this population would be resistant to these antimicrobials. Nonetheless, other studies in the same districts revealed the presence of isolates resistant to third-generation cephalosporins in poultry. For example, Hamisi *et al*. [22, 28] found 29.8 % of poultry isolates were resistant to cefotaxime, whereas Rugumisa *et al.* [25, 29] observed a lower prevalence of ceftazidime resistance. Although the presence of imipenem and ceftazidime on farms is unlikely to be associated with their direct use in poultry, our results suggest that bacteria resistant to these antibiotics can be found in other local reservoirs [26]. Bacteria with *bla*TEM and *bla*CTX-M79 genes have been reported in closed (i.e. tap water) and open water sources in the northern part of Tanzania, indicating the existence of alternative AMR bacteria reservoirs in lakes and rivers [26]. Considering most farmers in the northern zone of Tanzania use tap water for poultry production, the presence of *bla*TEM genes and *bla*CTX-M79 in tap water may explain ceftazidime resistance in *E. coli* isolates from animals that were not exposed to antimicrobials [26]. Prior to this study, no research into imipenem resistance in poultry had been conducted in Tanzania. As a result, no direct evidence could be found indicating the origins of imipenem resistance in poultry. Despite restrictions on imipenem usage in Tanzania, informal use may occur due to limited regulatory enforcement and access to antibiotics [30]. This informal practice can be driven by factors such as antibiotic availability without a prescription, self-medication culture and economic considerations in the poultry industry [30]. Animals can also acquire imipenem-resistant *E. coli* from humans via faeces if they are exposed to human excrement [28].

In the current study, poultry-derived isolates had smaller zone sizes than EUCAST reference isolates and were subsequently classified as resistant based on EUCAST thresholds. This highlights the potential for misclassification of a portion of poultry isolates from the normal distribution as resistant, according to the EUCAST reference distributions, and ECOFFS (which are primarily derived from human-centric datasets). Similar shifts have been observed when comparing EUCAST data to Gram-negative isolates from animals [18]. Humans and animals have inherent variability, which may explain why WT distributions considered normal in animals may not be normal in humans. Animals, including ruminants and other herbivores, have more complex digestive tracts than humans [30, 31, 32]. One key factor contributing to this variation is largely due to the diversity of microbes in the animal gut versus that of humans [33, 34]. The animal gut contains a wider variety of microbes that contribute to metabolic processes and nutrient breakdown [33, 34]. The pH of the gut, nutritional availability, diet and interactions with host factors can influence bacterial proliferation, including those that harbour antibiotic resistance genes [35]. In agricultural and veterinary settings, animals are often exposed to antimicrobial agents for therapeutic puposes, growth promotion or prohylaxis [30]. In the presence of this selective pressure, resistance to antimicrobials, including resistance caused by efflux pumps, can develop and spread. Furthermore, varying antimicrobial use patterns can contribute to differences in resistance profiles, as well as efflux pump expression. According to recent research, upregulated efflux pumps have been found to be prevalent in animals compared to humans [31]. The AcrAB-TolC efflux pump system, for instance, has been linked to multidrug resistance and frequently is upregulated in animal-associated bacteria, such as poultry [36]. On the other hand, the MexXY-OprM efflux pump system, which is frequently found in *Pseudomonas aeruginosa *, confers resistance to many antimicrobials, including flouroquinolones and aminoglycosides, and has been observed to be highly expressed in animal-associated strains [37]. Contrary to the underlying evidence supporting IZD variation, Sjölund *et al*. [38] found similar distributions between human and poultry isolates, despite poultry-derived isolates being collected from environments without antimicrobial exposure, thus suggesting that the two populations have similar WT (wild) populations. In contrast to what was found in this study, Sjölund *et al*. [38] revealed that wild-type bird isolates exhibited similar distributions to human isolates, despite being collected from birds in pristine environments with little exposure to antimicrobial agents [38]. Nevertheless, other reasons that may explain variations and decrepancies may have been attributed to methodology in these studies, despite systematic efforts to standardize procedures [12], Furthermore, EUCAST distributions are inherently known to originate from data generated by different sources [39]. Our observations, however, may be an artefact of the resistance mechanisms that might have developed as a result of antimicrobial exposure, and hence the shift to lower zones of inhibition.

Our study acknowledges the potential bias introduced by selecting microbes based on a single breakpoint at the beginning of our analysis where we implemented a screening approach using McConkey agar with and without antibiotics, categorizing isolates that grew on media with antibiotics as resistant. We recognize that including the three breakpoints would have provided a more comprehensive assessment of the impact of different breakpoints on resistance classification. However, due to technical limitations, we were unable to incorporate that at the beginning of our study. Despite this limitation, our study still provides valuable insights by focusing on the comparison of specific breakpoints and their implications in resistance classification. By investigating these selected breakpoints, we shed light on their specific characteristics and provide meaningful insights within the defined scope of our study

## Conclusion

This study illustrates how different thresholds can impact the interpretation of resistance. ECOFFs or CB thresholds may overestimate [40] or underestimate prevalence when used instead of bespoke thresholds. EUCAST is largely composed of the human-centric datasets SENTRY and MYSTIC, with little representation of African and animal data. As a consequence, the EUCAST dataset does not accurately portray the WT distribution of human or poultry *E. coli* isolates from Africa. Additionally, clinical breakpoints are developed for human therapeutic purposes, but the same breakpoints are applied to interpret information from various animal studies. Considering limitations of datasets used to generate the thresholds, it is uncertain whether the existing threshold schemes are true universal reference metrics for resistance interpretation, since they may lead to misinterpretations of resistance, particularly in low-income countries. It is important to re-examine the current thresholds and include data from low-resource countries to make the thresholds more inclusive.

## Supplementary Data

Supplementary material 1
